# European cooperation for clinical practice guidelines in cancer

**DOI:** 10.1038/sj.bjc.6601076

**Published:** 2003-08-15

**Authors:** T Philip, B Fervers, M Haugh, R Otter, G Browman

**Affiliations:** 1 FNCLCC, Paris, France; 2 Centre Léon Berard, Lyon, France; 3 University of Groningen, Groningen, The Netherlands; 4 McMaster University and the Hamilton Regional Cancer Centre, Hamilton, Canada

**Keywords:** practice guidelines, international co-operation

All countries in the European Community face common challenges for delivering appropriate and high-quality care to patients with cancer. However, despite tangible improvements in diagnosis and treatment, marked differences in cancer survival across European regions exist (Sant *et al*, 2001).

The translation of existing research results into consistent patient-oriented strategies in the form of evidence reviews is a key endeavour to help improve cancer care and patient outcomes. Clinical practice guidelines (CPGs) are important tools for informing health-care professionals and patients about appropriate clinical practice in cancer care. Numerous guidelines development programmes have been set up in most European countries to develop CPGs in various specialities, including oncology (Courté-Wienecke *et al*, 1999).

However, recent studies have shown that published guidelines vary in quality (Shaneyfelt *et al*, 1999; Grilli *et al*, 2000; Graham *et al*, 2001) and some may even recommend suboptimal care. It has been established that it is essential that CPGs are evidence-based, so as to provide a valid tool for those caring for patients with cancer. To be credible, CPGs should also be developed with the input of local multidisciplinary cancer specialists (Ray-Coquard *et al*, 1997,Ray-Coquard *et al*, 2002) to encourage local acceptance and use in clinical practice.

In addition to the process of development, guideline ‘after-care’, i.e. the combination of dissemination, implementation and guideline updating, has been recognised as being important to the success of guidelines (Browman, 2001a,Browman, 2001b). Some oncology CPGs have been shown to lead to significant changes in practice when dissemination and implementation are conceptualised from the beginning of the guideline development process (Ray-Coquard *et al*, 1997,Ray-Coquard *et al*, 2002; Fervers *et al*, 2001b).

The development, updating and implementation of high-quality CPGs require substantial resources, both in terms of time and money. Currently, there is no systemic and structured approach to the development, evaluation and monitoring of guidelines at the international level. Moreover, there is no infrastructure to sustain and further develop these efforts. As a result, efforts are unnecessarily duplicated by organisations in different countries aiming at similar goals and using similar strategies. This may result in the wastage of financial and structural resources, and the suboptimal management of activities. These resources include the time volunteered by experts who participate in the working groups.

European countries could benefit from a multinational European collaboration for guidelines development, to minimise costs and avoid existing unnecessary duplication of effort, and to improve the dissemination and implementation of CPGs that comply with internationally accepted quality criteria.

If a multinational collaboration for CPG development is to be set up, it should be built on existing CPGs initiatives involved in the development of CPGs in oncology. However, the CPGs initiatives in different countries use different methodological approaches. These differences can be explained, partly, by the differing reasons for guideline development and the characteristics of organisations involved in the CPG development process (Burgers *et al*, 2003). One major difference, for example, is the choice between a ‘top down’ strategy, organised by national (or regional) agencies, and a ‘bottom up’ strategy, based on professional initiative. Based on this diversity, the experiences of the various initiatives are heterogeneous, as shown by an international survey of 19 guidelines programmes. This situation yields a series of challenges that should be addressed and also raises questions that must be answered when setting up a multinational collaboration.

To ensure that CPGs will contribute to the improvement of care for cancer patients, CPG development and the reporting of that process should meet specific quality criteria (Grol *et al*, 2003). The different dimensions of guideline quality that have been developed and validated recently by the Appraisal of Guidelines, Research and Evaluation (AGREE) Collaboration (funded under the European Union Biomed II Research Programme) provide a framework for the development of guidelines at the international level (www.agreecollaboration.org). These dimensions include the rigour of the methods used to collect and synthesise the evidence, the process used to formulate the recommendations, the involvement of stakeholders and the applicability of the recommendations.

Quality assessment of guidelines using the AGREE instrument showed that CPGs produced as part of a structured programme had significantly higher quality scores than those published outside such programmes (AGREE Collaboration, 2003). As a consequence, multinational cooperative projects could provide appropriate infrastructure and the environment for sharing specific tasks in the CPGs development and updating processes as well as the dissemination and implementation processes. This includes methodological support at all levels and well-defined procedures for sharing responsibilities.

A systemic approach is the basis for identifying which aspects of the guidelines development process can be achieved in common on a European or international level, and which aspects require specific national and local input. Literature searching, critical appraisal and synthesis of the evidence are key elements in the guideline development and updating processes. They are also the most costly and time-consuming steps in the path from research results to evidence-informed recommendations. Although some of the key elements in the guidelines development and updating process should give consistent results, the cultural diversity among European countries, particularly in terms of the structure and organisation of care, can lead to legitimate variability in guideline recommendations (Fervers *et al*, 1997; Eisinger *et al*, 1999). This includes variations due to cost-considerations in reimbursement strategies and policy-making decisions (Mille *et al*, 2000).

The collaboration between the guidelines project ‘Standards, Options and Recommendations’ (SOR), run by the French National Federation of Cancer Centres (FNCLCC) (Fervers *et al*, 2001a), the OECI (European Organisation of Cancer Centres) and the Cancer Care Ontario Guidelines Initiative (CCOPGI) (Browman *et al*, 1995) has provided good insight into the challenges encountered when different countries cooperate. In particular, the collaboration between the SOR and the CCOPGI has helped our understanding of the legitimate reasons for discrepancies between guideline recommendations based on the same evidence, due to national and medical cultural differences (Browman, 2001a; Fervers *et al*, 2001b; Burgers *et al*, 2003). However, further research is needed to understand and to develop an explicit approach for consideration of cultural and contextual considerations in the formulation of the recommendations.

**Figure 1:**
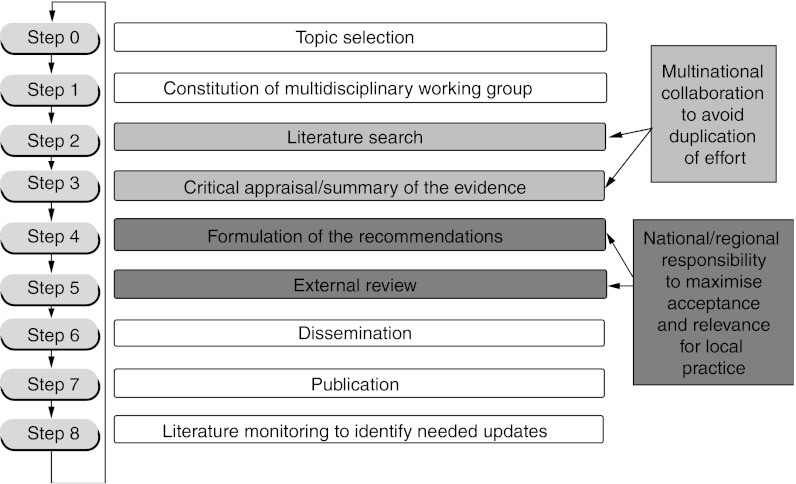
Framework for shared development of evidence-based clinical practice guidelines with local relevance. Reprinted with permission from Elsevier (The Lancet Oncology 2003; 4; 139–40)

Multinational cooperation should ensure that shared guidelines are relevant to national and local situations. This is crucial for the successful implementation of the guidelines and can be achieved by formulating the recommendations under national or local responsibility. The involvement of national and local stakeholders in the field of cancer from the participating countries will contribute to the enhancement of the local pertinence and acceptance of shared CPGs. The cultural diversity in Europe highlights the need for a transparent and explicit guidelines development process, in particular how recommendations are produced, with a clear link between the evidence and the recommendations, and information on how practitioners and other stakeholders are involved in the process. A process based on the Guidelines Development Cycle published by Browman *et al* (1995) provides a means of achieving both generalisable evidence-based recommendations and local relevance for practice (Figure 1

).

To achieve the objectives of CPGs, multinational collaboration should ensure accessibility of CPGs through appropriate dissemination and implementation strategies and by using different formats. These strategies should be tailored to the different target audiences involved in medical decision making (clinicians, policy makers, patients and other citizens) and clinical settings in the participating countries.

These observations, including the unnecessary duplication of effort among existing guideline initiatives, have led to an examination of how a multinational cooperative and structured approach to guideline development and updating, dissemination and implementation could be achieved in Europe (Fervers *et al*, 2002, http://eoi.cordis.lu/dsp_details.cfm?ID=29033). In Europe, as elsewhere, the reliability and sustainability of guidelines development programmes is an unquestionable challenge. To provide added value to existing guideline initiatives in Europe, the proposed multinational collaboration should aim to share existing experiences while taking into account the cultural and organisational diversity of the participating organisations and countries. This is important if national relevance and acceptance by guideline users are to be achieved, and if shared CPGs are to influence clinical practice. This has been shown to be crucial for the Canadian initiative when a national guidelines oncology programme for approval of new drugs was set up, involving the different provinces. In Europe, the different languages of the various European countries will add a level of complexity to this undertaking.

Promoting collaboration among guideline development initiatives in Europe will involve learning how we can build on existing initiatives and how we can adopt common standards to improve the consistency and quality of the guidelines. We will also have to learn how to share CPG development and updating responsibilities at an international level, and how to identify which responsibilities should be delegated to the national and local levels. Also, we need to learn how to increase the national relevance and local acceptance of shared guidelines, how to identify appropriate mechanisms for collaboration and how to provide an appropriate organisational framework for the shared development and updating of CPGs. The process will also involve identifying mechanisms for the development of related information for patients and their families, cancer advocacy groups and other citizens.

A proposal to set up a multinational collaboration for the development of cancer clinical practice guidelines is currently being elaborated. An outline proposal was submitted to the recent call for ‘Expression of Interest’ that was launched by the European union in the setting of its Sixth Framework programme for funding research (http://www.cordis.lu/fp6/). This collaboration will involve some members of the AGREE collaboration, a network of health services, research institutions and guidelines development organisations from 17 countries in Europe, USA, Canada and Asia-Pacific countries, and some of the partners in a network of European organisations working in the field of cancer, cancer centres (via OECI), professional societies and cancer-care specialists. This proposed multinational collaboration will make a significant contribution to avoiding duplication of effort and ensuring the availability of high-quality CPGs. This, in turn, will give individual health professionals better access to modern standards of clinical practice and thus contribute to improving care for cancer patients. Enabling patients and other citizens to understand cancer and its treatment better will improve their quality of life and help them to learn to live better with cancer.

In addition, multinational collaboration based on these principles will provide an excellent opportunity to explore the influence of culture and values in the path from scientific evidence to the final recommendation, and ultimately to patterns of practice. This will lead to a better understanding of the key elements in the guideline development process, and the impact of social, ethical and cultural issues on the effective dissemination and use of CPGs.
